# Examples of Gibsonian Affordances in Legged Robotics Research Using an Empirical, Generative Framework

**DOI:** 10.3389/fnbot.2020.00012

**Published:** 2020-02-20

**Authors:** Sonia F. Roberts, Daniel E. Koditschek, Lisa J. Miracchi

**Affiliations:** ^1^Department of Electrical and Systems Engineering, University of Pennsylvania, Philadelphia, PA, United States; ^2^Department of Philosophy, University of Pennsylvania, Philadelphia, PA, United States

**Keywords:** robot, affordance, legged, generative, reactive

## Abstract

Evidence from empirical literature suggests that explainable complex behaviors can be built from structured compositions of explainable component behaviors with known properties. Such component behaviors can be built to directly perceive and exploit affordances. Using six examples of recent research in legged robot locomotion, we suggest that robots can be programmed to effectively exploit affordances without developing explicit internal models of them. We use a generative framework to discuss the examples, because it helps us to separate—and thus clarify the relationship between—description of affordance exploitation from description of the internal representations used by the robot in that exploitation. Under this framework, details of the architecture and environment are related to the emergent behavior of the system via a generative explanation. For example, the specific method of information processing a robot uses might be related to the affordance the robot is designed to exploit via a formal analysis of its control policy. By considering the mutuality of the agent-environment system during robot behavior design, roboticists can thus develop robust architectures which implicitly exploit affordances. The manner of this exploitation is made explicit by a well constructed generative explanation.

## 1. Introduction

Gibson (1979) describes an *affordance* as a perceptually reliable feature of the environment that presents an agent with an opportunity for purposeful action. Autonomous robotics research can then be conceived as the process of designing a robot to systematically exploit the available opportunities for action in order to accomplish a specified overall task or tasks. In a complex, uncertain, and changing world, it is not obvious what design strategies will be most effective. It is therefore of general interest to ask how the explicit study of affordances might facilitate robotics research, and what the empirical study of affordances involves.

Ecological psychologists and philosophers have in general argued that perception and exploitation of affordances need not require the construction and manipulation of complex internal representations. Here, a “representation” refers to a model used in the control policy. For example, while a reactive controller could be argued to in some way represent the problem it is designed to solve, the system itself only senses and represents the variables used for feedback control. Beginning with the observation that agents have bodies (Chiel and Beer, 1997; Shapiro, 2011) and environments (Clark and Chalmers, 1998; Wilson and Golonka, 2013), and considering affordances to be properties of the embodied agent-environment system (Stoffregen, 2003; Chemero and Turvey, 2007), the argument is that the agent already has all of the action-relevant properties of the world available to it. It thus does not need complex internal representations, either of the world or of the affordances it exploits.

Others—even those sympathetic to the cause of embodied cognition (Clark, 1997, 1998) — have argued that complex representations are necessary for basic cognitive skills. It is elementary that some representation of internal state is necessary to achieve feedback stabilization of many control systems, and more comprehensive analyses suggest that robustness to complex perturbations in a complex world requires an agent to have complex internal representations of the environment and the perturbations (Conant and Ross Ashby, 1970; Francis and Wonham, 1976; Wonham, 1976). The role of internal models for limbs and their environmental loading in animal motor activity is widely accepted by many neuroscientists (Kawato, 1999). Deep learning (LeCun et al., 2015; Goodfellow et al., 2016) offers a modern version of this argument: The complexity of the problem a neural net can solve is in many ways limited by the size of the net and the amount of data, and thus the complexity of the model the net can build.

However, taking an ecological view, it may instead be the case that the agent needs only representations of “essential variables” which describe the relevant features of the activity the agent is involved in Fultot et al. (2019). Representations using only these essential variables may be of much lower dimensionality than representations of the full system. Seemingly complex behaviors can then be built from structured compositions of such simpler controllers operating on the variables essential to their component behavior. The behavior resulting from such compositions of simpler controllers has the added benefit of increased explainability (Cohen, 1995; Samek et al., 2017; Gunning and Aha, 2019) since the expected behavior of the component parts is known and, when properly conceived, their composition follows formal mathematical properties (Burridge et al., 1999; De and Koditschek, 2015).

Legged locomotion is one example of such a behavior. Robots are able to locomote using both very strong feed-forward control methods, such as central pattern generators (Saranli et al., 2001; Ijspeert et al., 2007; Ijspeert, 2008) as well as more distributed control (Cruse et al., 1998; Owaki et al., 2013; Owaki and Ishiguro, 2017; De and Koditschek, 2018) and many roboticists employ combinations thereof (Espenschied et al., 1996; Merel et al., 2019). Legged animals appear to pragmatically combine feedforward (Grillner, 1985; Whelan, 1996; Golubitsky et al., 1999; Minassian et al., 2017) and feedback (Pearson, 1995, 2004; Steuer and Guertin, 2019) controllers, implemented physically in both the mechanics of the body and in the nervous system (Cruse et al., 1995, 2006; Jindrich and Full, 2002; Sponberg and Full, 2008).

Research on insect navigation provides some support for the potential robustness and flexibility of coordinated systems of simple controllers, even for behaviors that had been previously believed to require cognitive maps (Tolman, 1948). Despite compelling evidence of complex cognition in invertebrate species (Jacobs and Menzel, 2014), recent studies of insect navigation (review: Wystrach and Graham, 2012) suggest that insects integrate information from multiple simple navigation systems (Hoinville and Wehner, 2018). These could include a path integration system (Wehner and Srinivasan, 2003); visual cues, with motivation controlling the switch between relevant cues (Cruse and Wehner, 2011); reactive collision avoidance (Bertrand et al., 2015); and a highly conserved systemic search mechanism when other mechanisms fail (Cheng et al., 2014). Each of these simple mechanisms for navigation operates in a relatively decentralized manner and requires little internal representation. Properly coordinated, together they are sufficient to produce robust navigation.

The literature on vertebrate navigation suggests that even animals which do seem to build complex spatial maps of their environments for navigation using special neural structures (O'Keefe and Burgess, 1996; Hafting et al., 2005; Savelli and Knierim, 2019) still rely on the coordination of multiple navigation systems (Moser et al., 2008)—perhaps one solving the problem of local navigation with landmarks, and the other providing directional bearing (Jacobs and Menzel, 2014). It has even been suggested that primates navigate available affordances and choose between them in virtue of neurally implemented competing feedback controllers (Cisek, 2007; Pezzulo and Cisek, 2016).

However, the robotics research often considered to exemplify the application of ecological concepts like affordances to robotics is generally either oriented toward building representations of affordances (Şahin et al., 2007; Zech et al., 2017; Andries et al., 2018; Hassanin et al., 2018) or toward biomimetic implementations of specific animal behaviors or capabilities (Beer, 1997; Cruse et al., 1998; Webb and Consilvio, 2001; Ijspeert, 2014) which then act as models for testing biological hypotheses (Webb, 2001, 2006). When non-biomimetic robotics research oriented toward coordinating simple controllers is considered (e.g., Braitenberg, 1986; Brooks, 1986; Raibert, 1986; Arkin, 1998), it is inevitably decades old. This motivates a reconsideration of how affordances are—and can be—studied in robotics, one that examines how simpler component processes and behaviors might be coordinated to explainably produce more complex and robust affordance exploitation.

We find the application of Miracchi (2017, 2019)'s *generative framework* useful for this discussion. The generative framework is designed to separate descriptions of a target behavior (in our case affordance exploitation) from commitments about the details of robot morphology, programming, or environment necessary to accomplish the task. The framework consists of three parts. The *basis* model describes aspects of the robot, including its methods of information processing, and relevant properties of the environment. The *emergent* model specifies the target behaviors in the relevant contexts. These behaviors operate at a larger spatiotemporal scale than those of the basis model, characterizing more global patterns and abstracting away from the details of implementation. Emergent behaviors are thus likely not to be obvious consequences of the basis model. The *generative* model specifies how features of the basis model determine (“generate”) features of the emergent model, thus explaining the emergent behavior in terms of the implementation details at the basis level^1^ (see Table 1).

**Table 1:** Definitions of terms used to describe the case studies under the generative framework. An example application to a simple reactive controller represented with a dynamical system is provided in parentheses.

**Term**	**Definition**
Basis model	Describes relatively more concrete aspects of the robot and environment relevant to the target behavior (e.g., equations of motion)
Emergent model	Describes relatively more abstract behavior qualifying as systemic, effective affordance exploitation (e.g., fixed point location)
Generative model	Formal analysis linking features of the basis and emergent models (e.g., stability analysis of fixed point)
Gibsonian affordance	An opportunity for action in an agent-environment system (emergent-level property)
Reactive control	Responsive to robot-environment system's state, with little or no memory
Parallel composition	Controllers operating simultaneously in the same basis level, interacting according to formally described rules
Sequential composition	“Chains” of controllers, with the successful execution of one sub-behavior setting up the next sub-behavior
Hierarchical composition	Controllers operating at different levels of abstraction, e.g., on a single limb, coordination of limbs, center of mass trajectory, or to set a global goal

We can demonstrate this separation of the basis and emergent models with an example application to two types of feedback controller. Say we have a proportional-derivative (PD) controller on the speed of a steam engine that operates by powering a motor to open or close a valve based on a reading given by an electronic velocity sensor such as an inertial measurement unit (IMU). The emergent model would then be the stable operation of the engine at the set point speed. The basis model would then include the engine dynamics, the way it is influenced by powering the valve, and the properties of the velocity sensor. Formal analysis on the stability of the PD controller would constitute the generative model. With properly set gains and an assumption that the sensor is within a certain percentage error, the controller would be guaranteed to bring the engine to the set speed. Now say the speed controller used a Watt governor instead (Van Gelder, 1995), which raises and lowers the arms controlling the amount of steam allowed through the valve using centrifugal forces determined by the speed of the prime mover. The emergent model would be the same, but the basis model would substitute the governor's mechanical equations of motion for the electronic signal processing model of the IMU. Similarly, we can consider what representations are sufficient to effectively exploit the target affordance specified by an emergent model.

We apply this framework to six case studies of legged locomotion involving reactive controllers in order to demonstrate how these projects can be usefully conceptualized as designing robots to exploit affordances without complex internal representations. We choose work that uses reactive controllers to generate affordance exploitation, not only because they are often referenced in research on affordances in animals (see above), but also because robotics research has long demonstrated their utility. Reactive controllers respond to the state of the robot-environment system with little or no memory; are robust; typically require modest explicit internal world models, if at all; can often be formally analyzed with tools from dynamical systems theory; and—correctly designed and implemented—must indefatigably steer the coupled robot-environment system toward an appropriate goal. Such controllers can also be composed into more complex systems (Brooks, 1986; Arkin, 1998), though it is vital to the explainability of the emergent behavior that these compositions follow formal mathematical rules (e.g., Burridge et al., 1999). Compositions can be made in parallel (Raibert, 1986; De and Koditschek, 2015, 2018), in sequence (Lozano-Perez et al., 1984; Burridge et al., 1999; Burke et al., 2019a,b), and hierarchically (Full and Koditschek, 1999), which requires proper coordination of more primitive subcomponents whose isolated behavior and interactions are both mathematically understood. These interactions generate the required emergent behavior (Vasilopoulos et al., 2018a,b) (see Table 1).

The case studies are drawn from one research group that typically provides formal generative explanations, easing the application of the generative framework. Focusing on one group also enabled us to quickly and deeply examine the ways in which systems can be designed to exploit affordances at multiple levels of abstraction from implementation details without introducing many different kinds of research problems, and to discuss each project with the primary and senior authors to ensure that the researchers agreed with this characterization of their work. We anticipate that similar analysis would be interesting to apply to a variety of other robotics research programs (e.g., Hatton and Choset, 2010; Hogan and Sternad, 2013; Majumdar and Tedrake, 2017; Burke et al., 2019a,b).

## 2. Case Studies

The six examples in this section are arranged in order of abstraction from the physics governing the robot's limb-ground interactions. We will mention the roles of parallel, sequential, and hierarchical compositions of controllers where appropriate. A concise, technical summary is provided in Table 2. Readers interested in the more detailed analysis leading to the summarized formal conclusions can refer to the specific equations, theorems, and figures in the original research papers which are noted in the table. The *emergent* and *basis* models (EM, BM) refer to the target behavior and implementation details of the paper cited in the last column. The *generative model* (GM) describes the formal analytical link, a mathematical explanation of how the target behavior emerges from the basis model (Table 1). The *Gibsonian affordance* (GA) describes in functional terms the designers' selected opportunity for action provided by the interaction of the robot's physical implementation with its environment, and the *agent-environment interaction* (AEI) specifies the perceptually reliable information or energetic exchange between the robot and the environment that makes this affordance possible to exploit.

**Table 2:** Concise analysis of case studies under generative Gibsonian framework.

**Section**	**Emergent model**	**Basis model**	**Generative model**	**Gibsonian affordance**	**Agent-environment interaction**	**References**
2.1	Energy-efficient locomotion on sand (Figure 5)	Direct-drive robot legs on dry granular media (Equation 1)	Virtual damping in leg triggered by decompression reduces work transferred to media (Figure 9)	Robot energy retention as a function of media sensitivity to policy-selected foot intrusion velocity (Sec. I.b.2)	Dissipated power (work exchange rate between robot and media) arising from virtually damped foot velocity	Roberts and Koditschek, 2018
2.2	Energy-efficient standing on complex or broken ground (Equation 1)	Internal and external gravitational loading at joints of legged robot on fixed rigid substrate (Equations 22, 23)	Descent of jointspace energetic cost landscape by quasi-static feedback control (Equations 29, 31)	Efficient body pose as a function of descent-selected interaction between body morphology and local substrate geometry (Figure 1)	Landscape descent control computed from internal proprioceptive (actuator currents) sensing	Johnson et al., 2012
2.3	Predictable steady state body heading from gait-obstacle interaction (Figure 11)	Gait mediated yaw mechanics (Equation 15) induced by obstacle disturbance field abstraction (Equation 11)	Locked heading calculated from basis model equilibrium (Equations 25, 26)	Body heading as a gait-selected function of interaction between body shape and periodic terrain geometry (Figure 3)	Body torque perturbations induced by gait-selected obstacle disturbance field	Qian and Koditschek, 2019
2.4	Autonomous terrain ascent (Equation 15) avoiding disk obstacles (Equation 14) of sparse unknown placement	Point particle (Equation 35), or kinematic (Equation 44) and dynamic (Equation 51) unicycle mechanics with local range (Equation 26) and vestibular (Equation 55) sensing.	Global correctness for gradient-driven point particle abstraction (Thm. 3.2); more conservative guarantees for kinematic (Thm. 3.5) and dynamic (Thm. 3.9) unicycle	Safe reactive path to local peaks and ridges as an obstacle-policy-selected function of terrain slope (Figure 2)	Controller velocity or force commands driven by instantaneously sensed terrain slope mediated by obstacle-robot vector	Ilhan et al., 2018
2.5	Planar navigation to a global goal avoiding familiar complex obstacles of sparse unknown placement (Figure 1)	Point-particle (Equation 14) or kinematic unicycle mechanics (Equation 18) with global position sensor and obstacle recognition and localization oracle (Equation 12)	Global correctness of obstacle-abstraction controller for point-particle (Thm. 1) and kinematic unicycle (Thm. 2)	Safe reactive path to global goal as a function of memory-triggered obstacle abstraction policy (Figure 4)	Controller velocity commands driven by instantaneously sensed goal-robot vector mediated by obstacle abstraction	Vasilopoulos and Koditschek, 2018
2.6	Execution of deliberative assembly plan in planar environment (Figure 1) with sparse, unknown, complex, prox-regular (Def. 3) obstacles	Kinematic unicycle mechanics (Equation 1) with global position and dense local depth-map sensors (Equation 3)	Faithful assembly plan execution with obstacle avoiding excursions guaranteed to insure progress toward sub-goals (Thm. 1) modulo correct object manipulation modes (Sec. C.2)	Safe reactive paths to deliberatively sequenced sub-goals as a policy-selected function of obstacle boundary shapes (Figure 7)	Reference path tracking controller driven by path-error vector and obstacle boundary	Vasilopoulos et al., 2018a

*Where appropriate, we have specified the figures, theorems, and equations in the source material that correspond to the emergent, basis, and generative models, and the affordance exploited*.

### 2.1. Energetic Cost of Running on Sand

Roberts and Koditschek (2018, 2019) develop a reactive controller to reduce the cost of transport (EM) for a direct-drive robot with programmable compliance in its legs (Kenneally et al., 2016) locomoting on granular media (BM) using a combination of simulation and physical emulation experiments. Robust locomotion can be accomplished by a composition of decoupled controllers which individually affect vertical motion and the robot's directional velocity (Raibert, 1986).

When a robot pushes off of a compliant substrate like sand (Aguilar and Goldman, 2016), its foot, which has a smaller mass than the body, can quickly penetrate deep into the sand before the body begins to move up. Because the dissipation function of the sand is quadratic in velocity, the robot can transfer – and thus lose—a large amount of energy from its motors through its leg and foot to the ground while pushing off (GA). The energy wasted in transfer to the ground is significantly reduced by adding damping to the robot's leg “spring” in proportion to the vertical foot intrusion velocity (GM).

To implement Roberts and Koditschek's controller, the robot needs only the high-bandwidth information about its foot intrusion velocity provided by the direct-drive architecture and a measurement of the distance to the ground (AEI). It does not require an explicit internal representation of the ground (Hubicki et al., 2016).

### 2.2. Manipulating a Robot's Body Pose Using Its Limbs

Johnson et al. (2012) develop a controller that distributes effort between limbs of a six-legged robot standing on rigid, uneven terrain. Statically stable poses require the projected center of mass to lie within a polygon defined by the toes, which typically requires much more torque from some motors than others on uneven surfaces. Distributing effort between legs reduces the maximum torque requirement, lowering the overall energetic cost of standing and avoiding damage to the motors from overheating (EM).

To develop the controller, the authors build a “landscape” describing the energetic cost to stand as a function of body pose for a robot with a given legged morphology and a given toe placement (GA). They show formally that an effective descent direction toward a local minimum can be determined at every location on the landscape (GM) by the current draw from the motors, a direct measurement requiring no additional modeling (AEI).

Since the landscape can be expressed as the sum of costs due to the legs fighting each other in stance (internal forces) and the effort of the limbs to support the body mass (external forces), these two systems can be decoupled. The authors exploit this decomposition to implement the behavior on a physical six-legged robot as a parallel composition of two controllers: One to relax the internal forces by driving down the torque of legs operating in opposition to each other, and one to center the body mass over the toe polygon by driving down the body-averaged torques in parallel (BM).

### 2.3. Characterizing Interactions With Obstacles

Obstacles in a robot's environment could be used as opportunities for the robot to perturb its trajectory toward a desired direction. Qian and Koditschek (2019) walk a small robot with four legs and a fast-manufacturable body through a periodic obstacle field consisting of evenly spaced half-cylinders. By systematic experimentation, they observe the emergence of a yaw angle that locks the robot's steady-state trajectory over the obstacles in a manner relatively invariant to the robot's initial conditions upon entering the field (EM). This locked angle is an empirically stable function of body aspect ratio, the spacing between the obstacles, and the robot's gait (GA).

The authors develop an abstract representation of the effective yawing disturbance field resulting from the interaction between the robot's body aspect ratio and the spacing between the obstacles, which is a selectable consequence of gait (BM). The result is a dynamical model whose equilibrium states predict the resulting steady-state body yaw angle of the robot—and thus its steady-state locked heading (GM). The only feedback signals used by the robot are position and velocity measurements on the rotation of the legs, which are used for feedback control on the clock-driven position and velocity commands sent to the legs. These are sufficient to recruit the desired interaction between the body morphology and the environment structure, and the robot's heading stabilizes in absence of any body-level sensing (AEI).

### 2.4. Reactive Control on a Global Scale

Ilhan et al. (2018) develop a controller that drives a robot toward the locally most elevated position from any start location in a gentle hillslope environment punctured by tree-like, disk-shaped obstacles (EM). They test their controller on a physical six-legged robot walking on unstructured, forested hillslopes, using the top of the hillslope as the goal location. The robot uses an inertial measurement unit to acquire the local gradient, and a laser range finder to detect obstacles which are likely to be insurmountable (AEI).

A reactive “navigation”-level controller takes information about the local gradient and the presence of local obstacles, and produces a summed vector indicating the direction that increases the robot's elevation while avoiding a collision (GA). The coordination of the limbs to execute these commands is handled by a lower-level controller in a hierarchical composition.

The authors present multiple options of such compositions that assume different degrees of actuation authority for the lower-level controller. The strongest conclusions from formal analysis (global stability; GM-1) come from assuming the robot can be treated as a fully velocity-controlled two degree-of-freedom point particle (BM-1). More realistic models of outdoor mobility, which have more conservative guarantees (GM-2), assume that the robot can be treated as a non-holonomically constrained, velocity-controlled unicycle, or—when running at speed—a force-controlled unicycle.

### 2.5. Using Recognition of Complex Obstacles to Create Abstract Spaces Conditioned for Reactive Control Schemes

In contrast to the previous case study, which had a perceptually detected environmental feature as the goal location and a purely reactive control scheme, Vasilopoulos and Koditschek (2018) develop a controller that governs navigation in an environment with perceptually intricate obstacles toward an arbitrary, user-selected goal (EM). Obstacles may be highly complex, but if non-convex or densely packed then they are expected to be “familiar." The robot is assumed to have access to its global position and to an oracle with a catalog of non-convex obstacles on which it was previously trained (Pavlakos et al., 2017) (BM). When an obstacle enters the robot's sensory footprint at execution time, the obstacle can thus be instantaneously recognized and localized. Unrecognized obstacles are presumed to be convex and suitably sparse.

Once recognized, non-convex obstacles are abstracted to a generic round shape using a smooth change of coordinates, and if densely packed, may be conglomerated into one large obstacle. The result is a geometrically simple, abstracted space. A purely reactive navigation controller (Arslan and Koditschek, 2019) closes the loop to guarantee obstacle free convergence to the goal location within the geometrically simplified but topologically equivalent environment (GA). Actuation commands for the geometrically detailed physical environment with non-convex obstacles are obtained by pushing forward the abstracted navigation commands through this change of coordinates (AEI). The authors perform a formal analysis of the overall dynamical system describing the closed-loop controller navigating reactively through the abstracted space as it is updated by the robot's perception of new obstacles. Proofs of correctness (GM-1, GM-2) are provided for two actuation schemes: A fully actuated point particle robot (BM-1), and a kinematic unicycle (BM-2). In a recent extension (Vasilopoulos et al., under review), the composed controller is implemented on a physical legged robot, with the unicycle commands interpreted by a lower-level controller for the robot's gait in a hierarchical composition.

The contribution of this project may at first seem to be at odds with our interpretation of affordances, but we suggest that this project demonstrates how two very different methods can usefully complement one another. The problems of obstacle perception and navigation can be separated by the judicious use of a previously learned library of objects. This separation reduces the navigation problem to one which can be solved with reactive navigation control, about which formal guarantees can be provided. Methods like deep learning can then be used to produce the necessary library of objects. A careful composition of the navigation capability provided by the reactive controller and object recognition capabilities provided by the learning methods then produces the emergent behavior described in this project.

### 2.6. Layering Deliberative and Reactive Controllers

Vasilopoulos et al. (2018b) build on the previously described navigation system to develop a deliberative (offline) planning and reactive (online) control architecture which enables a robot to rearrange multiple objects in its imperfectly known environment. During execution of each deliberatively sequenced sub-goal, the combined controller produces reactive commands (GA) as a function of the recognized obstacle's boundary shape. Theoretical work (Vasilopoulos et al., 2018b) assumes a robot constrained to move as a kinematic unicycle, with a globally known position, and an omnidirectional LIDAR producing a dense local depth map (BM). Physical experiments (Vasilopoulos et al., 2018a) are performed with a four-legged direct-drive robot, with a hierarchically arranged composition of controllers coordinating the robot's legs to produce the kinematic unicycle-like behavior. The emergent model of the velocity-controlled unicycle is generated by a subcomponent basis model consisting of properly coordinated parallel and sequential compositions of hybrid Lagrangian stance dynamics (Topping et al., 2019). The velocity-controlled unicycle then becomes the basis model of the reactive controller in this project, producing the whole-robot behavior.

Formal generative analysis (GM) in these papers addresses only the interaction between the deliberative planner and the reactive controller. The former breaks down the task of moving multiple objects to multiple goals into an ordered set of subtasks assigned to the latter. The reactive controller, which is endowed with the same oracle as the previous case study, is able to drive the robot around unanticipated obstacles (AEI) as needed in order to execute subtasks as they are assigned by the deliberative symbolic controller. The robot is then able to grab and move each object toward its planned subgoal location (EM).

The use of a reactive layer to handle obstacle interactions significantly simplifies the control problem, and allows the authors to provide formal guarantees about the conditions under which this combined controller should be expected to succeed. The offline, symbolic deliberative layer effectively solves the abstracted task planning problem. High-level commands from this layer drive the reactive manipulation and navigation layer, which can use realtime signal processing and control to readily handle unexpected geometric and topological complexities which would seriously challenge a symbolic planner.

## 3. Discussion

A major contribution of engineering to the understanding of affordances more generally is the formal methods which are used to describe the generative relationships between the implementation details and the desired behavior. We hope to encourage crossdisciplinary interest in projects using these methods, and find the clear separation of the specified target behavior from the implementation details provided by a generative framework is helpful for discussion. We suggest that the use of affordances in robotics research need not include the development of computational models of those affordances for robots to identify and thus exploit. Instead, consideration of the mutuality of the agent-environment system during robot behavior design can be used to develop robust and explainable architectures which implicitly exploit affordances. Roboticists can and do use systematic, empirical practice to apply Gibson's philosophy of affordances—just without naming them.

Considerations of engineering design and the practicability of abstraction from the environment at different levels of planning and control can determine the mix of endowed prior knowledge, representation building, and sensory dependence. For example, with the last case study, we suggest that methodological commitment to use only reactive controllers (as by e.g., Brooks, 1986, 1991) distracts from the potential benefits of combining a focus on affordances during robot behavior design with a cautionary approach to internal representations^2^. If effort need not be spent to create representations that are useful for the robot to perform its basic behaviors like locomotion and navigation, then the effort can be spent to create useful representations for tasks which do require them, such as to enable better communication between collaborating robots and humans. For example, a team of robots tasked with helping geomorphologists study erosion in the desert might build a map of the ground stiffness in different locations (Qian et al., 2017). Such a map would be useful even if the robots are able to navigate and locomote completely with reactive control, which allows each robot to continue functioning normally even when it loses signal connection to team members, damages an end effector, or experiences a sensor glitch. Why not reserve the difficult task of building good representations for behaviors that require them, and use affordance-based reactive control for behaviors that don't?

## Author Contributions

SR and LM linked the generative framework developed by LM to the literature on embodied cognition and artificial intelligence. SR and DK linked the embodied cognition and control theory literatures. SR, LM, and DK applied the framework to the case studies. SR worked with primary authors to ensure that their approaches and results were appropriately characterized.

### Conflict of Interest

The authors declare that the research was conducted in the absence of any commercial or financial relationships that could be construed as a potential conflict of interest.

## Footnotes

^1^(Miracchi, 2019) uses the term “agent” model because of her focus on agency and intentionality. Here we use the term “emergent” both for increased generality and because we do not wish to take a stand on whether the target behaviors are agential in any important sense.

^2^As formalized by (Şahin et al., 2007), this is similar to taking the “observer" perspective rather than the “agent” perspective.
